# Therapy for persistent hypercalcemic hyperparathyroidism post-renal transplant: cinacalcet versus parathyroidectomy

**DOI:** 10.1590/2175-8239-JBN-2019-0207

**Published:** 2020-07-27

**Authors:** Gabriel Giollo Rivelli, Marcelo Lopes de Lima, Marilda Mazzali

**Affiliations:** 1 Universidade Estadual de Campinas, Faculdade de Ciências Médicas, Departamento de Clínica Médica, Campinas, SP, Brasil.; 2 Universidade Estadual de Campinas, Laboratório de Investigação em Transplante, Campinas, SP, Brasil.; 3 Universidade Estadual de Campinas, Faculdade de Ciências Médicas, Departamento de Cirurgia, Campinas, SP, Brasil.

**Keywords:** Hyperparathyroidism, Hypercalcemia, Hypophosphatemia, Parathyroidectomy, Cinacalcet, Kidney Transplantation, Hiperparatireoidismo, Hipercalcemia, Hipofosfatemia, Paratireoidectomia, Cinacalcete, Transplante de Rim

## Abstract

**Background:**

Persistent hyperparathyroidism post-transplant is associated with increases in the incidence of cardiovascular events, fractures, and deaths. The aim of this study was to compare both therapeutic options available: parathyroidectomy (PTX) and the calcimimetic agent cinacalcet.

**Methods:**

A single center retrospective study including adult renal transplant recipients who developed hypercalcemia due to persistent hyperparathyroidism. Inclusion criteria: PTH > 65 pg/mL with serum calcium > 11.5 mg/dL at any time after transplant or serum calcium persistently higher than 10.2 mg/dL one year after transplant. Patients treated with cinacalcet (n=46) were compared to patients treated with parathyroidectomy (n=30). Follow-up period was one year. Clinical and laboratory data were analyzed to compare efficacy and safety of both therapeutic modalities.

**Results:**

PTX controlled calcemia faster (month 1 x month 6) and reached significantly lower levels at month 12 (9.1±1.2 vs 9.7±0.8 mg/dL, p < 0.05); PTX patients showed significantly higher levels of serum phosphate (3.8±1.0 vs 2.9±0.5 mg/dL, p < 0.05) and returned PTH to normal levels (45±51 pg/mL). Cinacalcet, despite controlling calcium and phosphate in the long term, decreased but did not correct PTH (197±97 pg/mL). The proportion of patients that remained with PTH above normal range was 95% in the cinacalcet group and 22% in the PTX group. Patients treated with cinacalcet had better renal function (creatinine 1.2±0.3 vs 1.7±0.7 mg/dL, p < 0.05).

**Conclusions:**

Surgical treatment was superior to cinacalcet to correct the metabolic disorders of hyperparathyroidism despite being associated with worse renal function in the long term. Cinacalcet proved to be a safe and well tolerated drug.

## RESUMO

**Introdução:**

O hiperparatireoidismo persistente pós-transplante está associado a aumento na incidência de eventos cardiovasculares, fraturas e óbitos. O objetivo deste estudo foi comparar as opções terapêuticas disponíveis: paratireoidectomia (PTX) e o agente calcimimético cinacalcete.

**Métodos:**

Estudo retrospectivo de um único centro incluiu pacientes transplantados renais adultos que desenvolveram hipercalcemia devido a hiperparatireoidismo persistente. Critérios de inclusão: PTH > 65 pg/mL com cálcio sérico > 11,5 mg/dL a qualquer momento após o transplante, ou cálcio sérico persistentemente superior a 10,2 mg/dL um ano após o transplante. Os pacientes tratados com cinacalcete (n = 46) foram comparados aos pacientes tratados com paratireoidectomia (n = 30). O período de acompanhamento foi de um ano. Dados clínicos e laboratoriais foram analisados para comparar a eficácia e a segurança de ambas as modalidades terapêuticas.

**Resultados:**

a PTX controlou a calcemia mais rapidamente (mês 1 x mês 6) e atingiu níveis significativamente mais baixos no mês 12 (9,1 ± 1,2 v.s. 9,7 ± 0,8 mg/dL, p < 0,05); pacientes submetidos à PTX apresentaram níveis significativamente mais altos de fósforo sérico (3,8 ± 1,0 v.s. 2,9 ± 0,5 mg/dL, p < 0,05) e retornaram aos níveis normais de PTH (45 ± 51 pg/mL). O cinacalcete, apesar de controlar o cálcio e o fósforo no longo prazo, diminuiu, mas não corrigiu o PTH (197 ± 97 pg/mL). A proporção de pacientes que permaneceram com PTH acima da faixa normal foi de 95% no grupo cinacalcete e 22% no grupo PTX. Os pacientes tratados com cinacalcete apresentaram melhor função renal (creatinina 1,2 ± 0,3 v.s. 1,7 ± 0,7 mg/dL, p < 0,05).

**Conclusões:**

O tratamento cirúrgico foi superior ao cinacalcete para corrigir os distúrbios metabólicos do hiperparatireoidismo, apesar de estar associado a pior função renal no longo prazo. Cinacalcete provou ser um medicamento seguro e bem tolerado.

## Introduction

Renal transplant is the treatment of choice for end-stage chronic kidney disease because it can reverse metabolic complications, improve quality of life, and increase patient survival^1^^-^ 3. With the improvement in the long term for patient and graft survival, new challenges have emerged in clinical practice, focusing on the appropriate management of the most common chronic complications, such as vascular calcification, cardiovascular disease, and bone disease resulting from persistent hyperparathyroidism.

Despite the recovery of renal function after successful renal transplant, 20 to 50% of patients present with persistent hyperparathyroidism at the end of the first year of follow-up, with a lower chance of spontaneous remission afterwards^4^^-^ 7. The maintenance of elevated parathormone (PTH) levels leads to hypercalcemia, hypophosphatemia, and reduced bone mass with increased risk of fractures, as well as vascular calcification, with an increased risk of cardiovascular events and graft dysfunction^8^.

The excess of PTH leads to hypercalcemia through the following mechanisms: increased intestinal calcium absorption, increased tubular calcium reabsorption, and increased bone reabsorption. The main causal factors involved in the genesis of hypophosphatemia are: decreased activity of the sodium-phosphate co-transporter in the proximal tubule by PTH action, leading to phosphaturia; dysfunction of proximal tubule; excess of FGF23; renal denervation; and calcineurin inhibitor tubulopathy^9^^,^ 10.

The aim of this study was to compare the safety and efficacy of two different therapies for persistent hyperparathyroidism: the calcimimetic agent cinacalcet and parathyroidectomy (PTX). There are no data available from Brazilian centers comparing both treatments. In our country, due to peculiarities of renal replacement therapy, we found patients with more severe hyperparathyroidism than that reported in other studies based on European and North American populations. These facts justify the study and show its importance.

## Patients And Methods

This was a single center retrospective observational study based on the review of medical records of adult renal transplant patients who presented with persistent hyperparathyroidism post-transplant (PTH > 65 pg/mL) and hypercalcemia, treated with cinacalcet, in a renal transplant program from a University Hospital in Campinas, São Paulo, Brazil, from 2012 to 2017. Data was compared with a historical control group of patients with persistent hyperparathyroidism and hypercalcemia treated with parathyroidectomy from 1999 to 2011.

Inclusion criteria for this study were: PTH > 65 pg/mL and presence of persistent hypercalcemia (serum calcium > 10.2 mg/dL) one year after transplant; or severe hypercalcemia (serum calcium > 11.5 mg/dL) any time after transplant; or symptomatic hypercalcemia; or impaired graft function associated with hypercalcemia and follow-up after intervention longer than 12 months. All patients included in the study had GFR > 30 mL/min/1.73m^2^.

Exclusion criteria were: absence of data for analysis; follow-up after intervention shorter than 12 months; GFR < 30 mL/min/1.73m^2^; and hypercalcemia due to other causes.

Demographic data included: age, gender, race, etiology of chronic kidney disease, length on renal replacement therapy, donor source (living or deceased), interval from transplantation to intervention, blood pressure, and incidence of comorbidities.

Laboratory data was collected at baseline (time of intervention), and after 1, 6, and 12 months and included: PTH (normal range 15-65 pg/mL - electrochemiluminescence method), serum calcium (normal range 8.8-10.2 mg/dL - colorimetric method), serum phosphate (normal range 2.5-4.5 mg/dL - photometric method), alkaline phosphatase (normal range 30-120 U/L - kinetic-enzymatic method), and serum creatinine (normal range 0.7-1.3 mg/dL - kinetic-enzymatic method).

Glomerular filtration rate was calculated at baseline and after 12 months, using the CKD-Epi formula, and reported as mL/min/1.73m^2^ 11.

For comparison, patients were divided into two groups, according to the following therapeutic schedule: cinacalcet group (baseline was defined as the beginning of drug therapy; the initial daily dose was of 30 mg p.o. and adjusted according to calcium levels or occurrence of adverse events) and parathyroidectomy group (baseline was defined as the day of surgery; the surgical protocol was total parathyroidectomy with implantation of a small portion of gland in the deltoid muscle).

## Safety And Efficacy Analysis

### Safety

Cinacalcet group: presence of adverse events (classified as mild/moderate when there was spontaneous resolution or improvement with symptomatic medications, or severe when dose adjustment or drug withdrawal was needed), need for drug withdrawal, variations in immunosuppressive drug levels, and episodes of acute rejection.

Parathyroidectomy group: surgical complications, length of stay after surgery, increase in baseline creatinine. Post-surgical hypoparathyroidism was defined as PTH levels < 10 pg/mL and need for prolonged therapy with calcium and calcitriol. 

### Efficacy

A reduction in calcium and PTH to normal levels were considered efficacy parameters for both groups.

### Statistical analysis

Continuous variables are presented as mean ± standard deviation, while categorical variables are reported as proportions. The Mann-Whitney test was used to compare numerical variables. The Fisher’s test was used to compare categorical variables. The sample had a normal distribution indicated by the Anderson-Darling test. Statistical significance was considered if p < 0.05. Data analysis was performed by the SAS System for Windows software, version 9.4.

## Results

Seventy-six patients fulfilled the inclusion criteria, forty-six from the cinacalcet group and thirty from the parathyroidectomy group. Both groups were comparable in most demographic parameters analyzed, except for donor source that included 14% of living donors in PTX group (Table 1).

**Table 1:** Demographic, clinical, and laboratory parameters from patients with persistent hyperparathyroidism post-transplant according to study groups.

	Cinacalcet	Parathyroidectomy	*p*
Number of patients	46	30	
Age (years old)	50 ±11	47 ± 9	0.32
Race (% white)	60%	70%	0.18
Gender (% male)	58%	50%	0.32
Length on dialysis (months)	67 ± 34	78 ± 35	0.37
Donor source (% deceased)	100%	86%	< 0.001
Interval from transplant to intervention (months)	37 ± 40	38 ± 31	0.18
Etiology of chronic kidney disease			
✓ Chronic glomerulonephritis	32%	30%	0.87
✓ Systemic Hypertension	28%	23%	0.50
✓ Others	40%	47%	0.39
Blood pressure (mmHg)			
✓ Systolic	133	136	0.13
✓ Diastolic	77	80	0.33
Comorbidities (%)			
✓ Hypertension	90%	92%	0.47
✓ Dyslipidemia	41%	45%	0.38
✓ Diabetes	22%	25%	0.34
✓ Coronary artery disease	34%	30%	0.35
✓ Smoking	19%	15%	0.42
Laboratory data at transplant			
✓ Serum calcium (mg/dL)	9.2 ± 0,8	9.3 ± 1,0	0.79
✓ Serum phosphate (mg/dL)	5.5 ± 1,8	5.6 ± 1,7	0.45
✓ PTH (pg/mL)	1271 ± 765	1298 ± 846	0.84
Imunossupression			
✓ Basiliximab induction	39%	86%	
✓ Thymoglobulin induction	61%	0%	
✓ No induction therapy	0%	14%	
✓ Tacrolimus / Cyclosporine	100% / 0%	55% / 45%	
✓ Mycophenolate / Azathioprine	86% / 14%	49% / 51%	
✓ Prednisone	100%	100%	

Normal range: calcium (8.8 - 10.2 mg/dL); phosphate (2.5 - 4.5 mg/dL); PTH (15 - 65 pg/mL).

### Efficacy analysis

**Figure 1:**
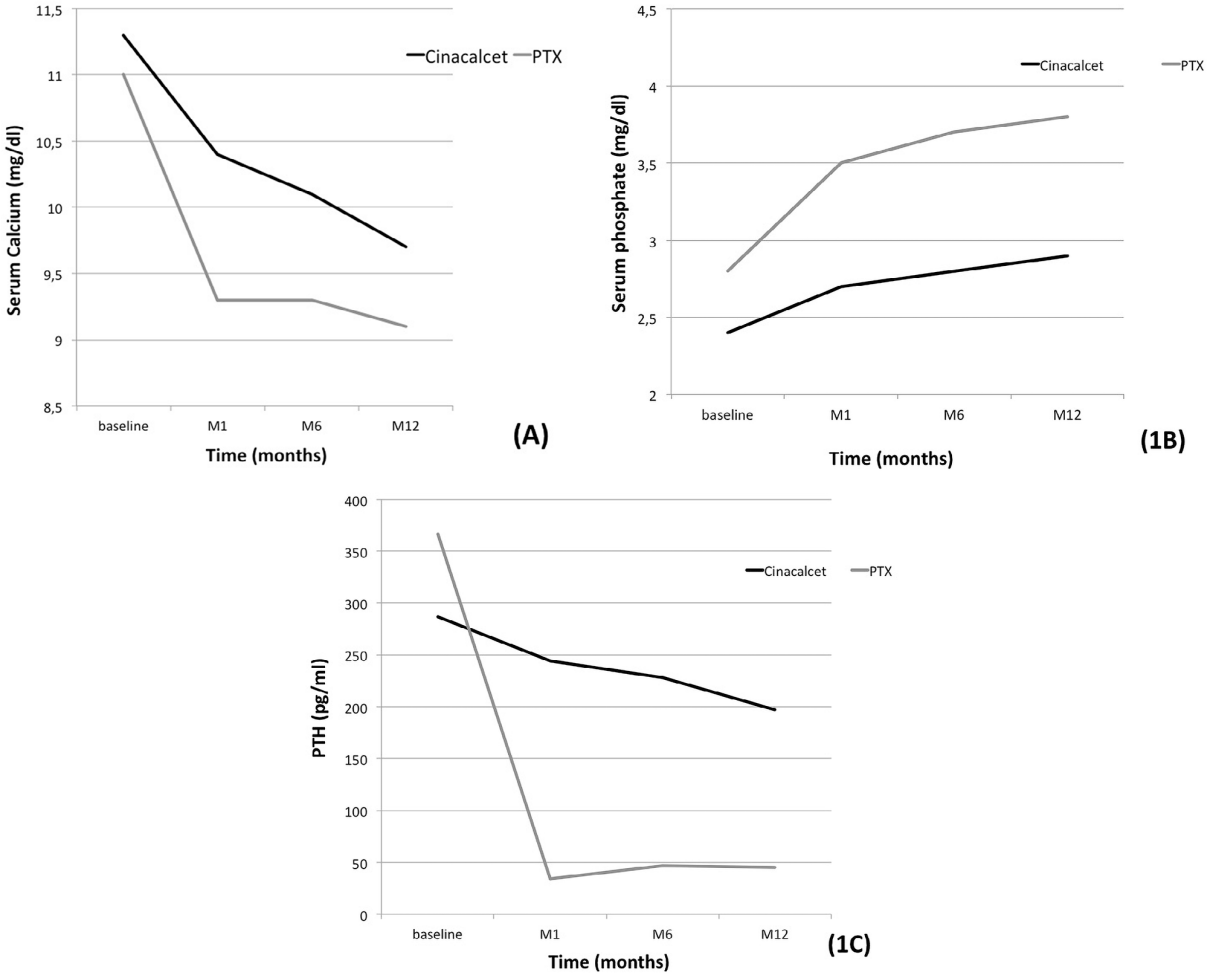
Laboratory data over the follow-up period. (A) serum calcium; (B) serum phosphate; (C) PTH.

**Figure 2:**
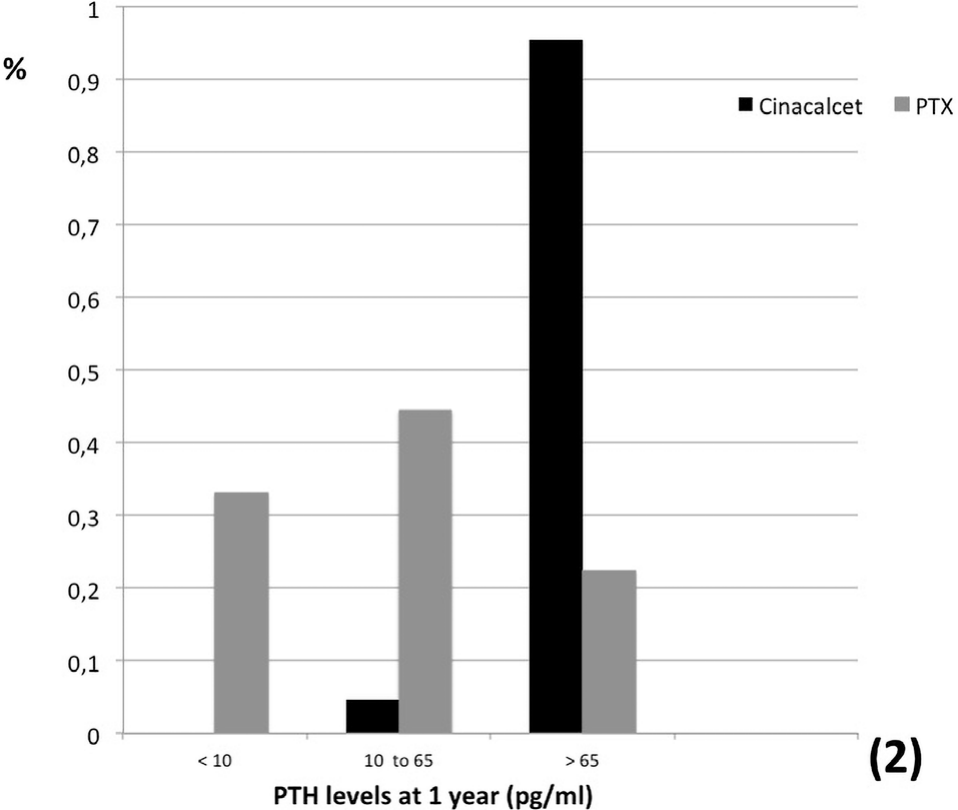
Gland function status at 1 year: hypoparathyroidism (PTH < 10 pg/mL), euparathyroidism (PTH at normal range) or hyperparathyroidism (PTH > 65 pg/mL).

Calcium levels returned to normal range faster in the surgical group with a progressive reduction over time, more significant in the PTX group (Table 2, Figure 1A). Normal calcium levels after one year of intervention were obtained in 74% of cinacalcet group compared to 92% in the PTX group (p=0.0011). Furthermore, we observed a progressive increase in serum phosphate levels over time, reaching normal range one year after transplant in 74% of the cinacalcet-treated patients compared to 96% of the PTX group (p<0.001) (Table 2, Figure 1B). While the reduction in PTH levels in the cinacalcet group was discrete, we noticed a marked reduction in PTH levels within the first month after parathyroidectomy (Table 2, Figure 1C). One year after the intervention, most patients (95.6%) in the cinacalcet group remained with PTH levels > 65 pg/mL compared to 22.3% in the PTX group (Figure 2). Renal function, measured by serum creatinine, remained stable over time in the cinacalcet group, while in the parathyroidectomy group we observed a stepwise increase in serum creatinine. Estimated glomerular filtration rate was significantly reduced in the PTX group one year after intervention (Table 2).

**Table 2:** Laboratory data for both groups over the follow-up period.

Parameters	Cinacalcet				Parathyroidectomy			
	Baseline	Month 1	Month 6	Month 12	Basal	Month 1	Month 6	Month 12
Serum calcium (mg/dL)	11.3 ± 0.7	10.4 ± 0.9* +	10.1 ± 0.8* +	9.7 ± 0.8* +	11.1 ± 1.1	9.3 ± 1.4*	9.3 ± 1.7*	9.1 ± 1.2*
Serum phosphate (mg/dL)	2.4 ± 0.6	2.7 ± 0.5* +	2.8 ± 0.5* +	2.9 ± 0.5* +	2.8 ± 0.7	3.5 ± 1.1*	3.7 ± 0.9*	3.8 ± 1.0*
PTH (pg/mL)	287 ± 160+	244 ± 115+	228 ± 133* +	197 ± 97* +	366 ± 144	34 ± 51*	47 ± 52*	45 ± 51*
Alkaline phosphatase (U/L)	108 ± 52	102 ± 52	97 ± 49* +	91 ± 39*	237 ± 375	182 ± 267	132± 246*	115 ± 188*
Creatinine (mg/dL)	1.2 ± 0.3	1.2 ± 0.3+	1.3 ± 0.3+	1.2 ± 0.3+	1.5 ± 0.5	1.6 ± 0.6	1.6 ± 0.6	1.7 ± 0.7*
CKD-Epi (mL/min/1.73m^2^)	64 ± 19			63 ± 18+	52 ± 17			46 ± 17*

* p < 0.05 versus baseline; + p < 0.05 between groups, for the same time point

### Safety analysis

Cinacalcet group: the initial dose of cinacalcet was of 30 mg p.o. and was adjusted to 60 mg/day within a 12-month intervention period. Fourteen patients (30%) reported some side effects, including nausea (15%), diarrhea (6%), vomiting (6%), vertigo (4%), and edema (2%). In some cases, these symptoms were controlled dividing the total daily dose of cinacalcet into two intakes. Ten patients needed 10 mg metoclopramide or 100 mg dimenhydrinate to relieve gastrointestinal side effects (nausea or vomiting). However, in three cases, cinacalcet was withdrawn because of severe gastrointestinal intolerance and the patients were referred for parathyroidectomy but their data after surgery were not included in this study. Additionaly, we did not observe acute rejection episodes or variations in immunosuppressive drugs levels during the study period. 

PTX group: mean hospitalization time after surgery was of 8 ± 3 days. The complications associated with the surgery included severe hypocalcemia with need for IV calcium correction (n = 10, 33%) and baseline creatinine variations (n =15, 50%). The majority of these cases (14 patients) presented with only a slight increase in serum creatinine. Only one patient required renal replacement therapy. This patient had a severe chronic graft dysfunction at the time of surgery, expecting to require hemodialysis in the short term. Surgical mortality was of 3% (n=1), as a consequence of massive cervical hematoma with airway compression, need for respiratory support, prolonged intensive care therapy, and death due to sepsis more than 3 months after the procedure. The incidence of post-surgical hypoparathyroidism with need for calcium and calcitriol prolonged therapy was of 56%.

## Discussion

Hyperparathyroidism is a frequent complication of chronic kidney disease and may persist after a successful renal transplant in 20-50% of patients^5^. The present study evaluated the safety and efficacy of treatments available to persistent hypercalcemic hyperparathyroidism: the calcimimetic drug cinacalcet *versus* parathyroidectomy in a group of adult renal transplant patients from a single center.

Hypercalcemia control was faster in the PTX group with normal range calcium and phosphate levels obtained within the first month after the procedure. Moreover, most patients had PTH levels lower than 65 pg/dL, classified as normal parathyroid function one year after the intervention. These results are comparable to a previous study by Meng et al.^12^ who reported a 100% rate of hypercalcemia control, normalization of serum phosphate, and reduction in PTH levels 12 months after PTX in a series of 15 patients. The preferred surgical procedure in our series was a total parathyroidectomy with partial gland reimplantation. This can explain the high incidence of severe transient hypocalcemia (33%) and definitive hypoparathyroidism (56%) with need for a calcium and calcitriol therapy, suggesting a high incidence of over-treatment. In another study, Evenepoel et al.^13^ reported an incidence of postoperative hypoparathyroidism higher than 50% in a retrospective study of 90 PTX after transplant. Further studies are needed to assess these outcomes with subtotal parathyroidectomy, which would reduce the incidence of post-surgical hypoparathyroidism. On the other hand, this technique could cause an increase in the incidence of persistent hyperparathyroidism post-PTX.

An increase in serum creatinine after surgery was observed in half of patients in the PTX group, which could have a negative impact in the long-term graft survival, with an increased risk for chronic allograft dysfunction. This is not an infrequent occurrence, as previous studies reported an incidence of worsening renal function after PTX ranging from 5 to 30%^13^^,^ 14, transient or permanent^15^^,^ 16. Worsening renal function was associated with worse graft function at the time of parathyroidectomy surgery^17^. Despite our high incidence of increased serum creatinine in the postoperative period, the vast majority of cases was manifested only by a slight increase in creatinine without major repercussions. PTH has a hemodynamic effect on the glomerular circulation. In an animal model, PTH was associated with a vasodilator effect on the afferent glomerular arteriole and vasoconstrictor effect on the efferent glomerular arteriole, leading to glomerular hyperfiltration^18^. After parathyroidectomy, there is a drop in glomerular filtration probably due to the loss of the positive effect of PTH on renal perfusion.

In recent years, the use of the calcimimetic cinacalcet appeared as an alternative to parathyroidectomy. In our series, we observed a progressive reduction in calcium levels, and after a one year follow-up, 74% of treated patients reached normal ranges. A similar response was observed regarding serum phosphate with a steady increase over time, reaching normal values after one year in 74% of patients. However, the reduction in PTH levels was slower, and most patients remained with PTH > 65 pg/mL at the end of follow-up. These findings are similar to previous reports in small series of transplant patients in whom a one-year therapy with cinacalcet was able to normalize both serum calcium and serum phosphate and reduce PTH levels but without achieving normalization^19^^,^ 20. The involution of the parathyroid gland hyperplasia after renal transplant is usually slow and, in some cases, can be partial^4^^-^ 6 ^,^ 21 ^,^ 22. Therefore, we expect that a longer therapy is needed with cinacalcet in order to obtain normal parathyroid gland function^23^.

Treatment with cinacalcet was well tolerated, with low incidence of side effects, mainly mild gastrointestinal symptoms. Withdrawal rate due to adverse events was low (6%), similar to previous studies^19^^,^ 23 ^-^ 26. Moreover, we did not observe acute rejection episodes or interaction with immunosuppressive therapy in this series. Renal function in the cinacalcet-treated patients remained stable over time, with a one-year filtration rate significantly superior to the PTX group. This protective effect of cinacalcet on renal function was also observed in previous reports^19^^,^ 20 ^,^ 26 ^,^ 27.

However, in relation to the renal function, it is important to point out that there was already a discrete difference in creatinine between groups at baseline (cinacalcet, 1.2 mg/dL x parathyroidectomy, 1.5 mg/dL). We believe that the main reason for this difference is the predominance of deceased donor organs in both groups. The quality of these organs depends on characteristics of the donor, such as age, comorbidities, baseline renal function, cause of brain death, and conditions of donor maintenance until nephrectomy. It also depends on post-nephrectomy factors such as the reperfusion solution and the total cold ischemia time, but these variables were not explored in our study. In addition, there may be some influence of the immunosuppressive regimen used by each patient. In the 1990s and early 2000s, our immunosuppressive protocol included azathioprine and higher doses of calcineurin inhibitors (cyclosporine), increasing its nephrotoxic potential. After the introduction of thymoglobulin as an induction drug in the protocol after 2010 and the switch from azatiprine to micophenolate, we started to use lower doses of the calcineurin inhibitors (tacrolimus) - a calcineurin-inhibitor minimization protocol.

There is little data comparing both treatments. A retrospective study by Yang et al.^24^, comparing 18 PTX with 13 patients treated with cinacalcet, showed earlier correction of hypercalcemia with PTX, with comparable calcium levels after one year. The authors reported persistence of 7% of hypercalcemia with cinacalcet, and renal function was similar between groups. Cruzado et al.^23^ conducted a prospective randomized controlled study comparing parathyroidectomy and cinacalcet treatments in 30 renal transplant patients with persistent hyperparathyroidism and hypercalcemia with 15 patients assigned to each arm. After one year, 100% of patients in the PTX arm had normocalcemia compared to 67% in the cinacalcet group whereas normal phosphate levels were obtained in 100% in the surgical group *versus* 93% in the cinacalcet arm. Of note, no patient in the cinacalcet group had normal levels of PTH, while 66% in the PTX reached normal parathyroid function. No change in renal function was observed in this series.

When a parathyroidectomy is performed, an important adaptative response to reduce renal function to maintain bone metabolism is lost. Viewed from this angle, calcimimetics are an interesting treatment option because they can achieve a more flexible and reversible hyperparathyroidism control. Nevertheless, the real role of cinacalcet in the treatment of persistent hyperparathyroidism after transplantation remains questionable: is it a palliative drug while waiting for a definitive treatment (parathyroidectomy), or does it have a real benefit in reversing gland hyperplasia? In favor of surgical treatment, a recently study demonstrated better long-term graft survival in patients treated with PTX compared to patients treated with cinacalcet^28^. Other previous studies demonstrated that cinacalcet withdrawal has been associated with a high incidence of recurrence of hypercalcemia^19^^,^ 22.

Previous studies with persistent hyperparathyroidism consider PTH at transplantation around 300 to 500 pg/mL, but in our series, 59% of patients in the cinacalcet group had severe hyperparathyroidism at transplant, with PTH levels ≥ 700 pg/mL. Despite this severity, results after a one-year therapy were similar to previous reports, suggesting that cinacalcet could be used as an alternative to PTX in these patients.

The limitations of this study are inherent to its retrospective design, with biases in patient selection and data collection in medical records. Through statistical analysis, we could verify that the groups were homogeneous and comparable, attenuating the selection bias. Additionally, this was a single center study but controlled by a group of patients treated with a standard therapy (parathyroidectomy). Sample size was small but comparable to previously published observational studies.

In conclusion, PTX proved to be more effective than cinacalcet to control metabolic disorders associated to hyperparathyroidism. Cinacalcet was well-tolerated, being a safe therapeutic option for renal transplant patients with hypercalcemic hyperparathyroidism, maintaining stable renal function. A longer follow-up period is necessary to observe the behavior of PTH with a prolonged cinacalcet therapy.
